# Rigid fusions of designed helical repeat binding proteins efficiently protect a binding surface from crystal contacts

**DOI:** 10.1038/s41598-019-52121-9

**Published:** 2019-11-07

**Authors:** Patrick Ernst, Annemarie Honegger, Floor van der Valk, Christina Ewald, Peer R. E. Mittl, Andreas Plückthun

**Affiliations:** 1 Department of Biochemistry, University of Zürich, Winterthurerstrasse 190, 8057 Zürich, Switzerland; 2 Present Address: Cytometry Facility, University of Zürich, Winterthurerstrasse 190, 8057 Zürich, Switzerland

**Keywords:** X-ray crystallography, Protein design

## Abstract

Designed armadillo repeat proteins (dArmRPs) bind extended peptides in a modular way. The consensus version recognises alternating arginines and lysines, with one dipeptide per repeat. For generating new binding specificities, the rapid and robust analysis by crystallography is key. Yet, we have previously found that crystal contacts can strongly influence this analysis, by displacing the peptide and potentially distorting the overall geometry of the scaffold. Therefore, we now used protein design to minimise these effects and expand the previously described concept of shared helices to rigidly connect dArmRPs and designed ankyrin repeat proteins (DARPins), which serve as a crystallisation chaperone. To shield the peptide-binding surface from crystal contacts, we rigidly fused two DARPins to the N- and C-terminal repeat of the dArmRP and linked the two DARPins by a disulfide bond. In this ring-like structure, peptide binding, on the inside of the ring, is very regular and undistorted, highlighting the truly modular binding mode. Thus, protein design was utilised to construct a well crystallising scaffold that prevents interference from crystal contacts with peptide binding and maintains the equilibrium structure of the dArmRP. Rigid DARPin-dArmRPs fusions will also be useful when chimeric binding proteins with predefined geometries are required.

## Introduction

**Figure 1:**
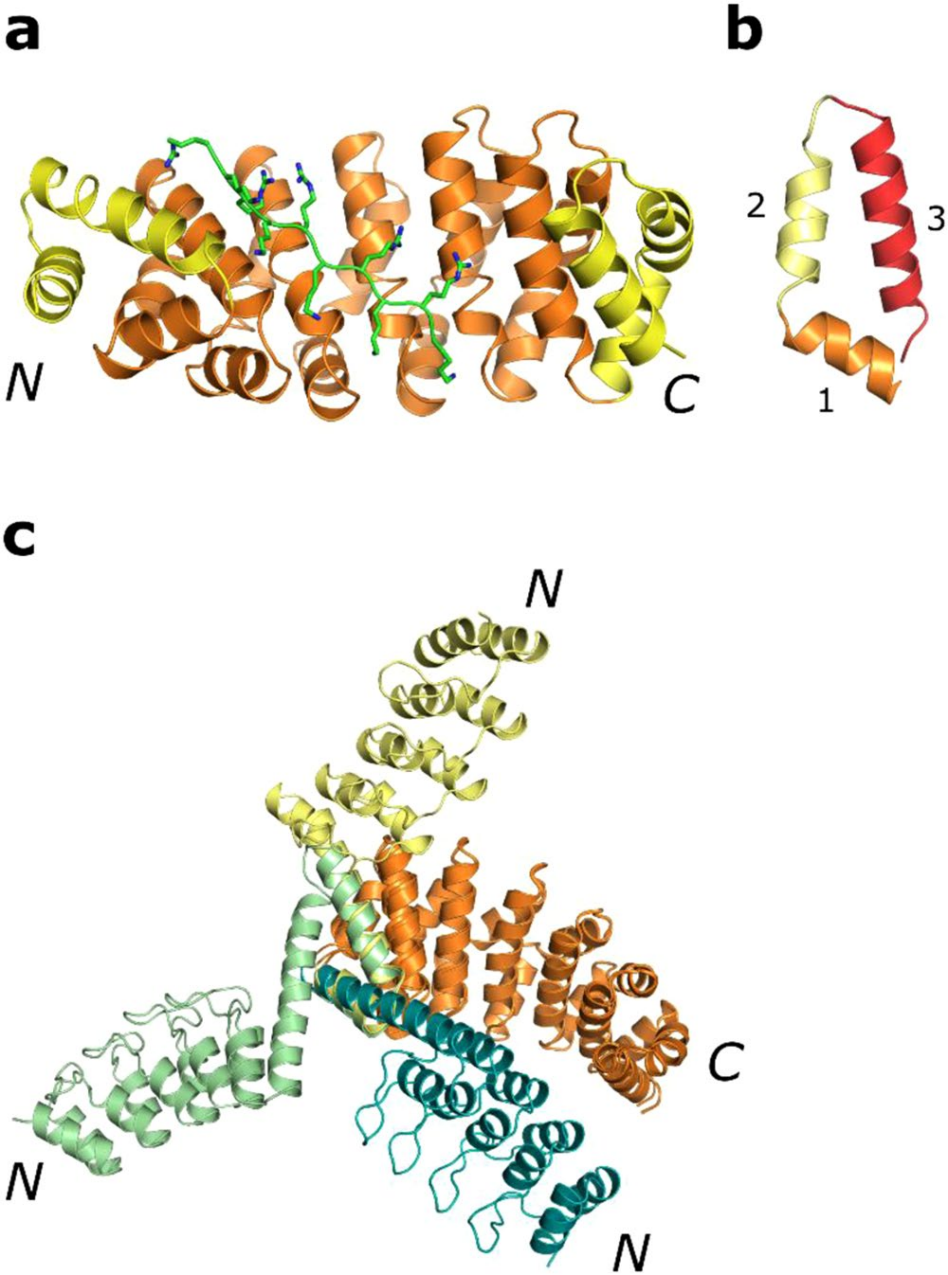
dArmRPs and DARPin-dArmRP fusions. (**a**) Crystal structure of a dArmRP with five internal repeats (orange), binding to a (KR)_5_ peptide (green) (PDB ID: 5AEI). The hydrophobic core is shielded by two capping repeats at the termini (yellow) from the aqueous environment. (**b**) Structure of a dArmRP repeat with helix H1 in orange, helix H2 in pale yellow and helix H3 in red. (**c**) Model of N-terminal DARPin-dArmRP fusions. The three different helices (H1, H2 or H3) of the dArmRP (orange) can be used to construct rigid DARPin fusions. Each of the fusions can be constructed with varying length of the helix, leading to a rotation of the fused DARPin around the shared helix as a function of helix length. The N-terminal capping repeat of the dArmRP is omitted in each case, as the shared helix is part of the first internal repeat. Three examples are shown with a fusion to helix one in teal, a fusion to helix two in light green and a fusion to helix three in pale yellow.

Designed Armadillo repeat proteins (dArmRPs) bind to elongated peptide sequences and may eventually complement classical detection antibodies (a review on alternative binding scaffolds can be found in ref.^1^, their use in therapeutics is reviewed in ref.^2^). dArmRPs are based on the helical natural armadillo repeat proteins (nArmRPs), an α-solenoid repeat protein family that binds to stretches of unfolded regions of proteins^3–5^. Over the last years, a monomeric and well expressing scaffold has been derived from ArmRPs by protein engineering^6–10^ (further reviewed in refs^11,12^). Stable and regularised repeats have been derived from the more irregular repeats of nArmRPs. In these proteins, a varying number of internal repeats, binding the peptide, and capping repeats, which shield the hydrophobic core from the aqueous environment, are stacked together to form a superhelical repeat protein (Fig. 1a). By stacking the internal repeats, or binding modules, a large concave and solvent-exposed binding surface is formed (Fig. 1a). Ideally, different modules, each recognising two side-chains of the peptide target, would be recombined to bind to arbitrary peptide sequences. These binding modules are obtained by preselection from libraries and/or protein design. In principle, these modules could be reassembled in any desired arrangement to bind new targets, thereby making costly individual selections against new targets unnecessary.

So far, modules binding to arginine- and lysine-rich peptides have been designed and the development of new binding pockets and modules is under way, both by selection techniques and by computational design methods (unpublished data). A key analysis technique for the design process is macromolecular crystallography, and numerous crystal structures have confirmed the sequence-specific binding of dArmRP to different target peptides^10,13–16^.

However, it soon became clear that crystal forces can have a large impact on the outcome and interpretation of experimental crystal structures^13,14,16^. By analysing 27 crystal structures of dArmRPs without peptide or in complex with their cognate target peptides, we found that, depending on the crystallisation conditions and crystal packing, the curvature of the superhelix, and consequently the peptide binding mode differed^16^. Moreover, we observed that in some crystals, peptide binding was no longer possible, since the specific packing did not sterically allow a peptide to be bound to the dArmRP, i.e., the formed crystal contacts displaced the peptide, despite its low nanomolar K_D_. Like other α-solenoid repeat proteins, dArmRPs frequently crystallise in a tubular arrangement. The packing of these tubules contributes to the curvature of the dArmRP, but the exact mode and magnitude of this effect remained to be clarified. Several other artefacts, like one peptide binding to more than one dArmRP in the crystal or register shifts of bound repetitive peptides have also been observed. In all of these cases, the intramolecular crystal packing on which crystallography relies disturbs or interferes with the desired molecular features. Since reproducible access to high resolution crystal structures is essential in developing the modular binder technology, we set out to solve this problem.

To validate our previous structures and to answer the question of the influence of the crystal lattice, we designed a crystallisation construct in which the influence of packing forces on the scaffold should be reduced. Ideally, the binding surface would be shielded to exclude any interactions other than the peptide binding to the dArmRP. For this purpose, we were aiming at generating a crystal structure with the longest so far crystallised continuous peptide binding surface and its cognate binding peptide, namely a dArmRP with six internal repeats binding the decapeptide (KR)_5_. Nearly all of the previously generated structures are dArmRPs with the same internal binding module but different repetitions and the six-repeat version would give us the most insight into continuous peptide binding.

Designed ankyrin repeat proteins (DARPins) are another class of helical repeat proteins which have been engineered as an alternative binding scaffold^17,18^. They are well expressing, thermostable and rigid, which is why they have been used as binding proteins in many different applications (reviewed in ref.^19^). In contrast to dArmRPs, DARPins preferentially bind to folded domains and carry a β-turn between helices that makes them very rigid. To broaden the scope of application for DARPins, they have already been extended in a rigid way by another fusion protein to provide additional crystal contacts. To achieve such fusions, the concept of rigid “shared helix” fusions of DARPins to other globular proteins or other DARPins was developed to facilitate crystallography of DARPin-target complexes^20,21^. The fused protein is connected to either the N- or C-terminal helix of the DARPin and provides an additional surface that can form crystal contacts. In the context of this work, it was observed that a specific DARPin, called D12, was particularly successful in providing conserved crystal contacts via its hydrophobic binding surface^21^. The fusion of D12 as a crystallisation chaperone has so far led to numerous crystal structures^21,22^.

Here we show how we applied rigid shared helix fusions and the well crystallising DARPin D12 to construct a crystallisation scaffold that exhibits ideal peptide binding on a dArmRP binding surface without interfering crystal contacts. Our designs are the first rigid fusions of two binding proteins with completely different binding modes. This work did not only answer questions regarding the design of dArmRP peptide binding surfaces but also demonstrates new possibilities for the construction of fusions of different types of binding proteins with a predictable orientation of the paratopes.

## Results

### Design of N-terminal DARPin-Armadillo fusion proteins

Shared helices between DARPins can be constructed by fusing DARPins at either the N- or C-terminal helix of the capping repeats. When this concept is expanded to dArmRPs, the potential number of fusion constructs increases, since one dArmRP module consists of three helices (H1, H2 and H3) and each helix can be used to create a shared helix fusion of variable length (Fig. 1b,c, SI Fig. 1). To validate that DARPin-dArmRP fusions can be generated as easily as DARPin-DARPin fusions, we constructed different fusions, initially concentrating on the construction of N-terminal fusions to limit the number of fusions to be tested. We fused the C-terminal helix of the DARPin C-cap to a helix (H1, H2 or H3) of the first internal repeat of the dArmRP, omitting the dArmRP N-cap. As a fusion partner we chose DARPin D12, which has been identified as a crystallisation chaperone, because of its ability to form many different sets of crystal contacts^21^. For this purpose, shared helices with varying length of 1–18 AA were designed *in silico* using the *Rosetta* protein design suite, as described in ref.^21^ and tested for clashes between the fused DARPin and dArmRP^21,23^. All shared helix designs that resulted in a direct clash between the DARPin and the dArmRP were excluded. Furthermore, the conserved crystal contacts that can be formed by DARPin D12 were taken into account with the intention to maintain them when constructing the fusions. To ensure reliable crystallisation we excluded shared helix designs in which the known contacts between the DARPin D12 paratopes would lead to clashes with other fusion proteins in the crystal lattice^21^. Finally, 16 potential fusion designs were identified and three constructs, each representing a fusion to one of the three dArmRP helices, were tested in crystallisation trials to validate that such fusions between DARPins and dArmRPs can be made.

### Crystal structure of a fusion of DARPin D12 to helix 2 of a dArmRP

**Figure 2:**
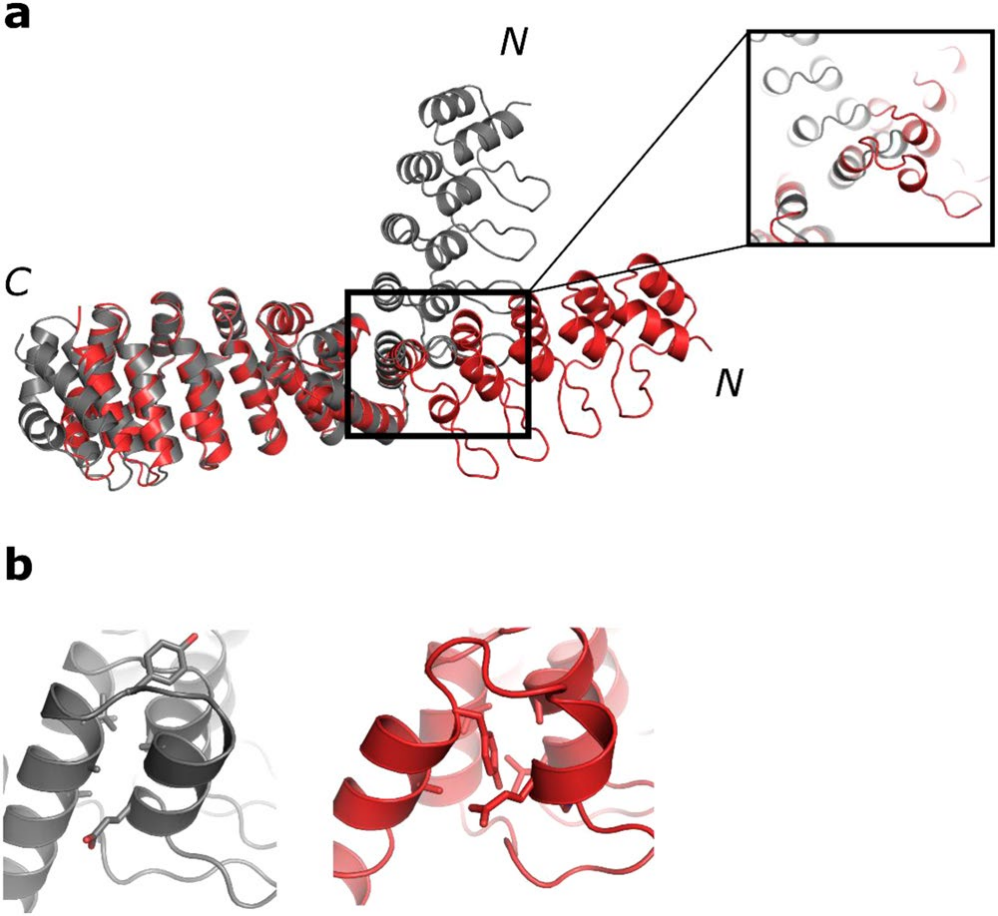
Comparison of model (grey) and crystal structure (red) of the fusion to helix 2 of the dArmRP. (**a**) Superposition of model (grey) and crystal structure (red, 1.6 Å resolution) of an N-terminal DARPin-dArmRP fusion. N- and C-termini of the proteins are marked. The fusion of DARPin D12 was made to H3 of a dArmRP with four internal repeats, with a shared helix length of 5 amino acids. The close-up view shows the distortion of the first helix of the DARPin. (**b**) Detailed view of the changed interface between model and experimental structure. The left picture is showing the model, the right one the structure in which Tyr150 inserts into the interface between the shared helix and the DARPin.

A fusion of the DARPin to H2 of the internal repeat of the dArmRP crystallized readily in space group P2_1_ and diffracted to 1.6 Å with one molecule in the asymmetric unit (Fig. 2). The crystal was densely packed with a solvent content of 41%. A crystal contact was found between the DARPin paratope and the dArmRP binding surface. The first helix of the DARPin (residues 145–148) was unwound and formed a longer loop instead of an α-helix. This was probably caused by both the crystal contact and its resulting forces as well as by an altered interface between the shared helix and the DARPin. Tyr150 pointed towards the interface and made key hydrophobic interactions, thus stabilising the changed interface, instead of lying on top of the interface as in the designed model (Fig. 2b**)**. On the other hand, the interface between the shared helix and the dArmRP aligned well with the design (Fig. 2a). Overall, this results in a Cα RMSD of 2.3 Å at the interface between the DARPin and the shared helix (residues 112–168) and in a Cα RMSD of 0.6 Å at the interface between the shared helix and the dArmRP (residues 150–208). This construct showed that our design strategy was applicable to fusions between dArmRPs and DARPins, however, crystal forces have to be taken into account as they can be strong enough to distort the interfaces of the shared helix. Although a target peptide was added in 1.5-fold molar excess prior to crystallisation, it was not visible in the electron density map. This was probably due to the crystal contact between a symmetry-related DARPin and the binding surface of the dArmRP, displacing the peptide. In summary, these results showed that further design efforts were required to prevent the blocking of the peptide-binding site by crystal contacts.

### Long and freestanding helices can be bent due to crystal forces

As the peptide was not visible in the first structure due to crystal contacts on the binding surface displacing the peptide, we constructed a new shared helix. This helix was different from the usual shared helix designs. So far, all shared helices were constructed such that the helix was stabilised by packing against the hydrophobic core of both fusion partners. However, to orient the DARPin above the binding surface of the dArmRP, a long and freestanding shared helix is required. Furthermore, we wanted to improve the occupancy and thus the electron density of the peptide bound to the dArmRP in the crystal. As we have shown previously^16^, flexible peptide linkers can provide an effective way to ensure peptide binding in the crystal. This resulted in a dArmRP design with six internal repeats, an N-terminally fused DARPin D12 and a C-terminally fused (KR)_5_-peptide.

**Figure 3:**
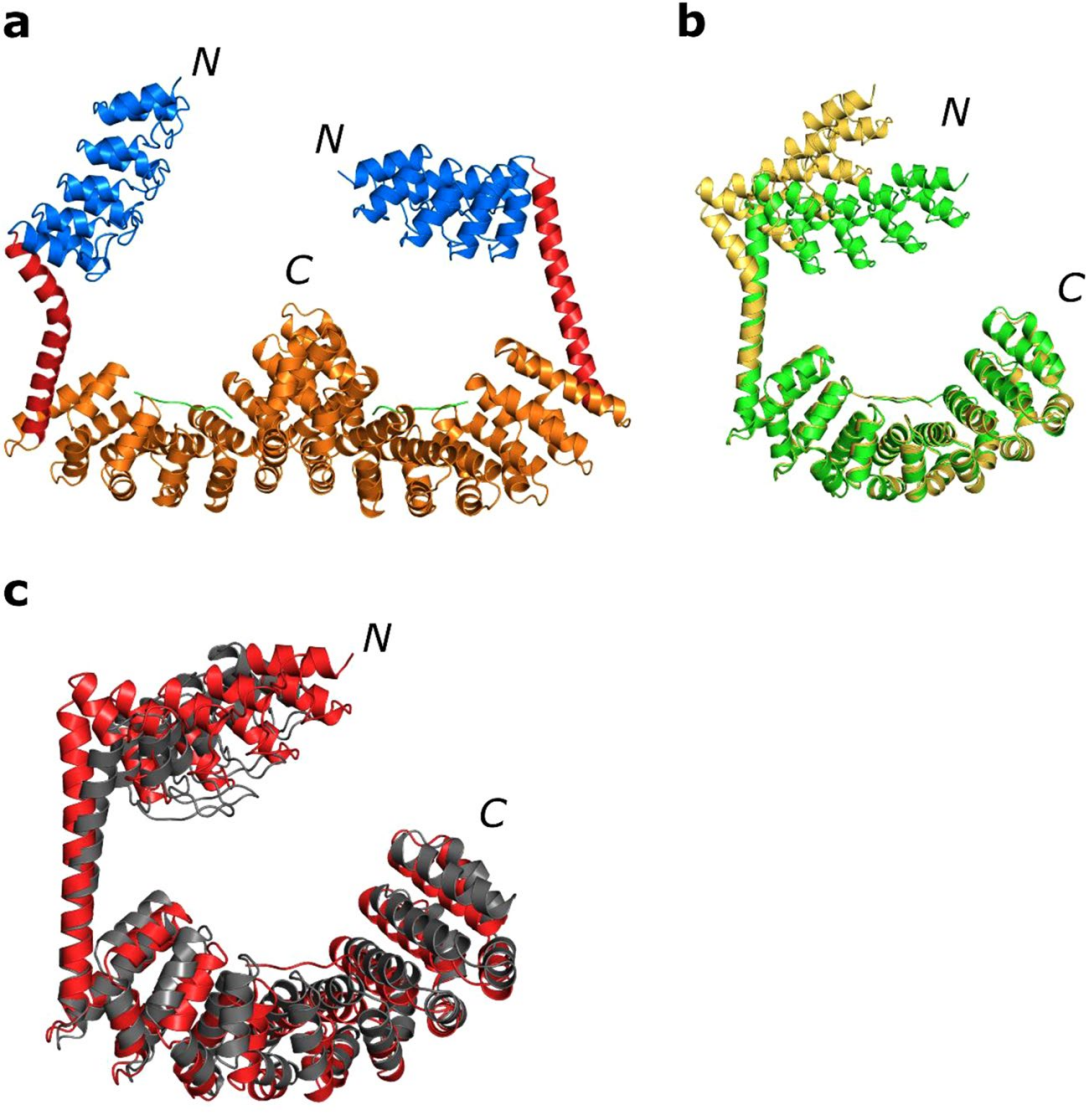
Crystal structure of the second generation construct with a longer shared helix at 3.3 Å. N- and C-termini of the proteins are marked. (**a**) Asymmetric unit with the DARPin in blue, the shared helix in red, the dArmRP in orange and the peptide in green. (**b**) Overlay of the two molecules of the asymmetric unit, one shared helix is bent due to crystal forces; chain A in green, chain B in yellow-orange. (**c**) Overlay of the designed model in grey with the crystal structure in red (chain A, residues 149–214 were used for the alignment).

The construct expressed well and crystallised in space group P2_1_ with two molecules in the asymmetric unit (Fig. 3a). The crystals of this rather bulky construct diffracted to 3.3 Å and had a high solvent content of 69%. The structure of the fusion showed that the chaperone structure was still suboptimal. The longer helix of one molecule in the asymmetric unit was straight (chain A), while the other was bent (chain B) and revealed that further design steps had to be undertaken to stabilise the shared helices (Fig. 3b,c). An alignment of residues 149–241 of the straight shared helix and the interface to the dArmRP to the design model resulted in a Cα RMSD of 3 Å for chain A and 3.9 Å for chain B. Nevertheless, the two molecules in the asymmetric unit showed a shielded binding surface without crystal contacts. Furthermore, electron density for the fused (KR)_5_-peptide was visible. The limited resolution, probably caused by the high solvent content and possibly imperfect packing due to the flexible shared helix, did not allow to reliably model the peptide in this data set. In order to reduce flexibility and further improve the construct we decided to fuse a second DARPin to the C-terminus of the dArmRP.

### Two fused DARPins at both dArmRP termini effectively shield the binding surface

To rigidify the construct and exclude potential crystal contacts to the binding interface, we constructed an additional C-terminal DARPin D12 fusion, again using the shared-helix strategy. The second DARPin was placed such that it came into close contact with the first DARPin, thereby creating a ring-like structure, which was further stabilised by a disulfide linker between the N- and C-terminal D12 DARPins (residues 10 and 625). The disulfide bond design was entirely based on an *in silico* model, situating 615 amino acids in between the two cysteine residues, since no prior crystal structure of a C-terminal dArmRP-DARPin fusion was available.

**Figure 4:**
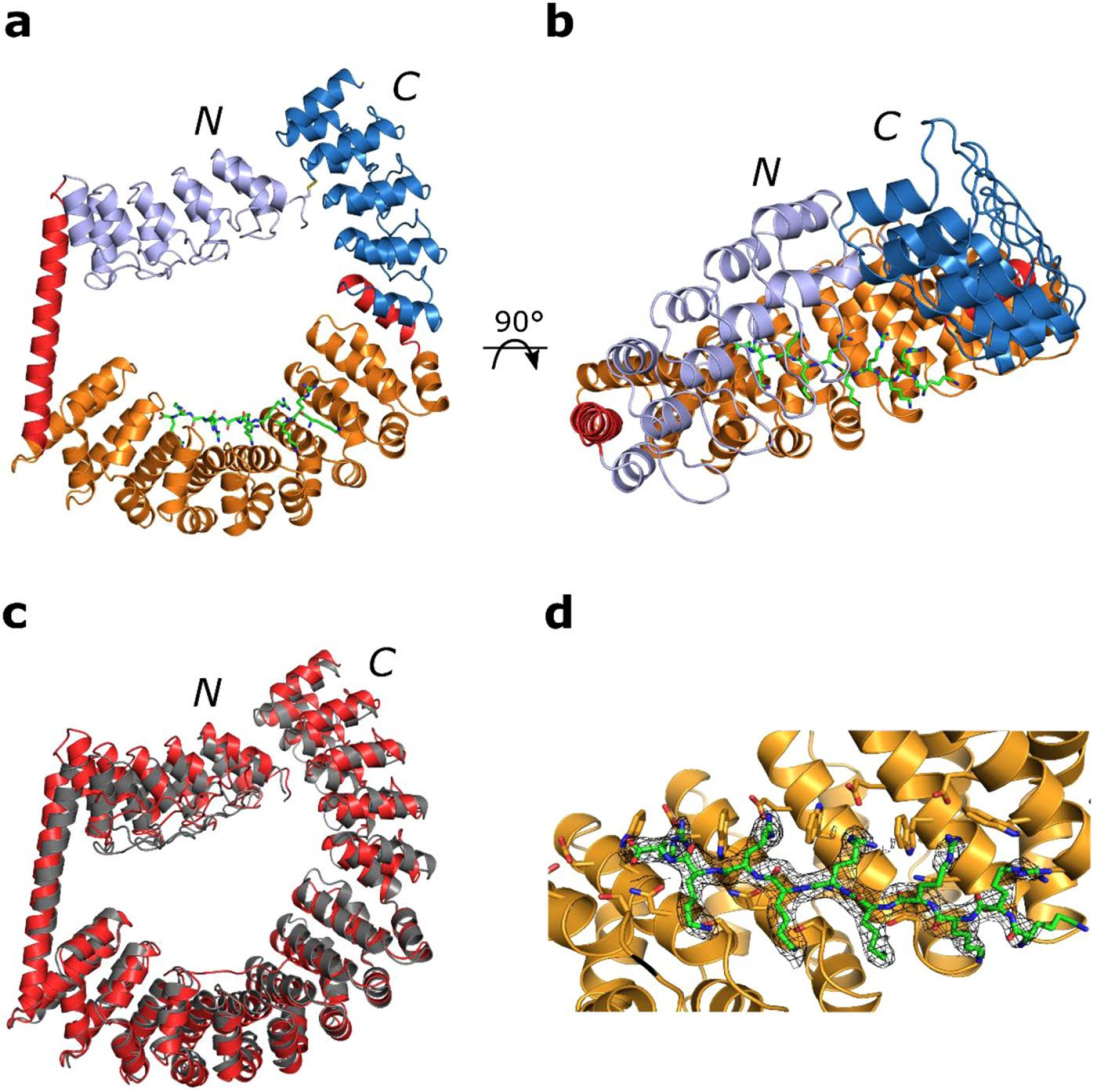
Crystal structure of the ring-like construct showing a fully shielded binding surface at 2.4 Å. N- and C-termini of the proteins are marked. (**a**) Asymmetric unit with the DARPins coloured in light blue (N-terminal DARPin) and marine (C-terminal DARPin), the shared helices in red, the dArmRP in orange and the peptide in green. (**b**) Top-view highlighting the shielded binding surface (an overview of the crystal packing is shown in Fig. 5). (**c**) Overlay of the designed model in grey with the crystal structure in red. (**d**) Close-up view of the binding surface showing the regular peptide binding. 2mF_o_-DF_c_ electron density of the peptide contoured at 1 σ.

The construct could be readily expressed and purified, and crystals grew after 6 days. They belonged to space group C222_1_ with one molecule in the asymmetric unit, and initial phases were obtained by molecular replacement using the designed structure as a search model **(**Fig. 4a,b). The first DARPin was hardly visible in the electron density and showed high B-factors, in contrast to the second DARPin, which was fused via a shared interface between the DARPin and the dArmRP. Yet, clear electron density was detected for the disulfide bond, which confirmed the accuracy of our designs. The overall Cα RMSD of the crystal structure to the design model was found to be 4.5 Å (Fig. 4c). The large deviations are a consequence of the elongated shape of the molecule. The DARPin domains are shifted in a distance-dependent manner from the dArmRP domain. During the full relax of the whole structure, which followed the side-chain mutation step in the *fixbb* run, the dArmRP domain became more compact than usually observed in crystal structures. While the Cα RMSD of the first DARPin and the shared helix (residues 7–190) to the design was 2 Å, the Cα RMSD of the second shared helix and the second DARPin (residues 485–671) to the design was only 1.7 Å.

**Figure 5:**
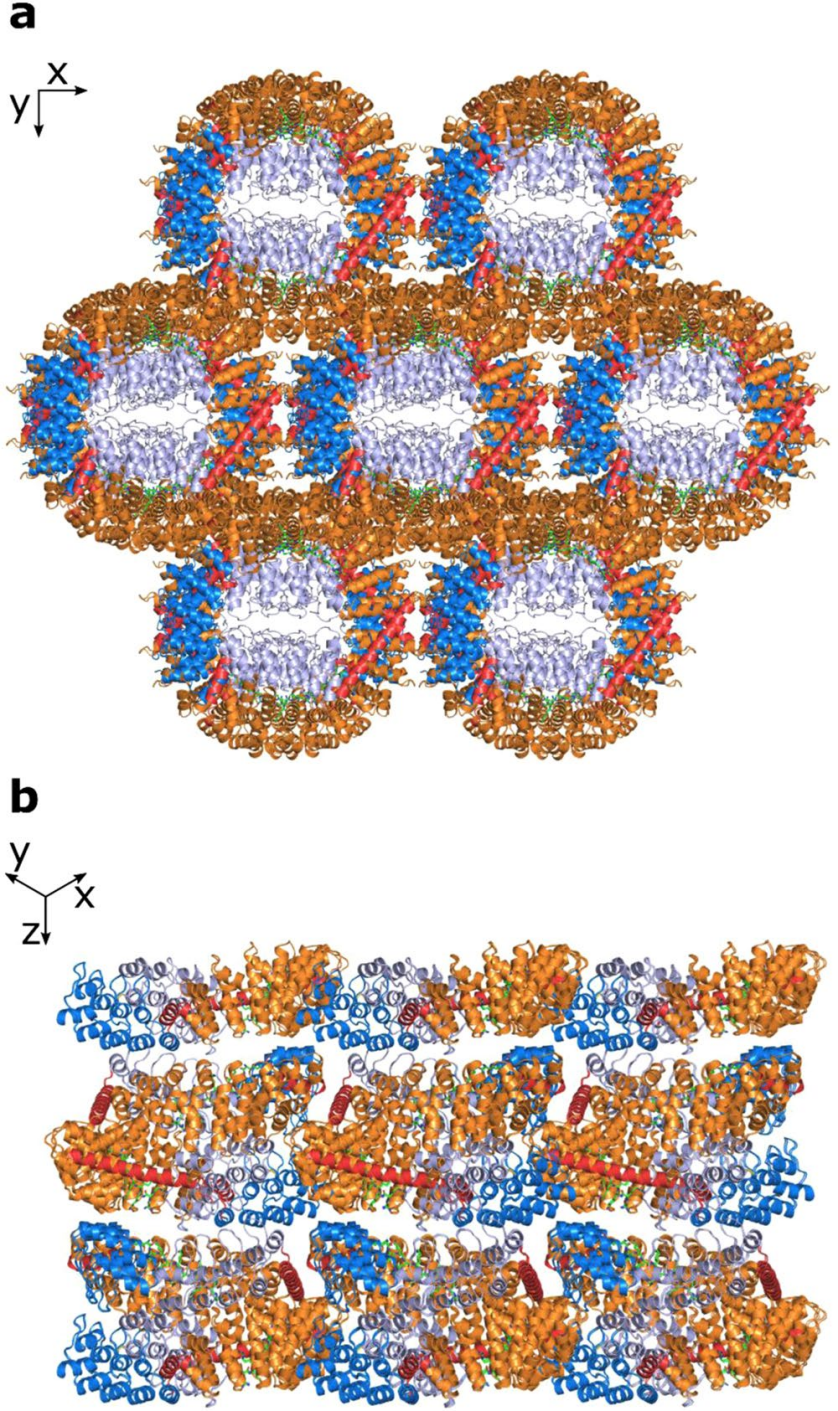
Crystal packing of the ring-like constructs. The DARPins are colored in light blue (N-terminal DARPin) and marine (C-terminal DARPin), the shared helices in red, the dArmRP in orange and the peptide in green. (**a**) View of the x/y plane, highlighting the tubular arrangement. (**b**) View in the plane of the z-axis with x and y at 45 degrees, highlighting the stacking of the rings forming the tubules.

With regard to peptide binding, the structure showed that the design process was successful and a fully shielded binding surface was obtained. One crystal contact was formed between the shared interface at the dArmRP and a symmetry-related shared helix, but no symmetry-related molecule did make a direct contact to the binding surface. Clear electron density was visible for the bound (KR)_5_-peptide, which was shifted by one repeat towards the C-terminus of the dArmRP on the repetitive surface (Fig. 4d, SI Fig. 2a). Residues 2–10 of the peptide bound very regularly and nearly identically in every binding pocket. Only the first arginine of the peptide did not bind in an ideal fashion due to the register shift. As expected from the design, every second peptide bond of the bound peptide was involved in bidentate hydrogen bonds to the conserved asparagine ladder, which keeps the peptide in an extended conformation (SI Fig. 2b). Interestingly, the construct crystallised again in a tubular arrangement with the rings stacking on top of each other, which would allow soaking of the target peptide (Fig. 5a,b).

### Shielded construct displays comparable curvature to previous structures

Our previous analysis of 27 dArmRP structures revealed that the curvature of dArmRPs is crucial for peptide binding^16^. The curvature, or supercoiling, can be defined by the distance and angles between two consecutive repeats^16^. As the peptide is bound in an extended conformation over the binding surface, the curvature has to match the backbone distance between peptide residues to allow for modular binding. With a maximal distance of 6.7–7 Å between the Cα atoms of the first amino acid and the (P + 2) amino acid in a stretched beta-strand conformation, the distance between two repeats also has to be in this range^16^. We concluded that the shielded construct exhibits an optimal curvature, since the binding of the (KR)_5_ peptide to the dArmRP was fully canonical and regular over the whole structure and extremely similar in each repeat. Table 1 shows the inter-repeat distances of the construct described here. Except for the first repeat, which was influenced by the above-mentioned crystal contact, the distances are close to the optimum and match the distances of previous structures that also showed an ideal peptide binding^16^. This also shows that ring formation did not disturb the structure, but on the contrary prevented its deformation by crystal contacts.

**Table 1:** Curvature parameters of the shielded crystallisation construct.

Repeat-pair	C_α_(P/P + 2) distance in Å
1–2	7.34
2–3	6.78
3–4	6.32
4–5	6.79
5–6	7.03

## Discussion

dArmRPs are currently being developed as modular peptide binders^11,12^. To achieve this goal, a variable number of internal repeats, or binding modules, with different binding specificities have to be stacked on top of each other to bind to arbitrary peptide sequences. In principle, any binder can be constructed from a subset of modules that have been selected beforehand for amino acid specificity. As soon as modules for most amino acids are available, there would be no need for any individual selection against the target peptide sequence to be bound.

For the development of this technology, a rapid feedback of both the binding performance and a verification of the exact binding mode and interaction with the pockets for the different side chains is required to guide the engineering and selection of the modules. Therefore, the analysis of developed modules is very critical, both for their binding properties and for the exact structural interactions of the peptide side chains with the pockets, and this will have to rely to a large extent on X-ray crystallography.

An analysis of 27 previously obtained dArmRP structures, most of them in complex with their target peptide, showed that crystal contacts can have a major impact on the binding of the peptide and the curvature of the dArmRP^16^. To achieve a reliable structural analysis feedback of different dArmRPs, we had to design constructs, which minimize the effect of crystal contacts on peptide binding or distortion of the dArmRP structure. Therefore, we wanted to create a crystallisation construct in which the binding surface of the dArmRP is fully shielded, thereby eliminating any undesired impact on peptide binding. For this purpose, we adapted the concept of rigid shared helices from DARPin-DARPin fusions to fusions between DARPins and dArmRPs^21^.

In a first round of design we evaluated whether rigid fusions between the well crystallising DARPin D12 and a dArmRP can be constructed in an analogous fashion to previously described DARPin-DARPin fusions^21^. A crystal structure of an N-terminal DARPin fusion to helix two of the internal repeat of a dArmRP was obtained. However, crystal contacts led to a distortion of the helical part of residues 145–148 and a different interface between the shared helix and the DARPin, involving a flip of Tyr150 into the interface. Distortion of the shared helix interfaces and deviations from the ideal design, potentially caused by crystal contacts, is a phenomenon we have previously observed in DARPin-DARPin fusions^21^. It has been shown that the formation of crystal contacts is favoured over keeping the intact conformation of the shared helices when DARPin D12, a very strong mediator of crystal contacts, is used^21^. Usually, these crystal contacts are observed between the binding paratopes of the DARPin; here we show that also secondary contacts to the dArmRP are strong enough to cause a similar distortion of the shared helices. Moreover, no peptide was visible in this crystal structure due to the crystal contact of a symmetry-related DARPin interacting with the binding surface of the dArmRP. In summary, DARPin D12 is such a good mediator of crystal contacts that regions within the remainder of the protein can be bent to accommodate them.

Because of the influence of the crystal contacts observed in the first construct mentioned above, we constructed a new fusion with an extended type of a shared helix. This shared helix was free-standing between the fusion partners and thus not involved in any kind of hydrophobic packing to the fused protein. The combination of this elongated shared helix with a peptide fusion of the target peptide to the dArmRP yielded the first structure of a dArmRP with a fully shielded binding surface. One limitation was that the shared helix was too flexible and bent in the structure. Furthermore, the crystal had a high solvent content of 69% and diffracted only to 3.3 Å. The electron density showed clear evidence for the presence of the peptide in the structure, although not sufficiently well defined at this resolution.

To overcome the limitations of the second construct, we expanded the shared helix approach of an N-terminally fused DARPin to additionally fuse a DARPin to the C-terminus of the dArmRP. This construct was further rigidified by adding a disulfide linker between the two DARPins. Importantly, this disulfide bond was placed entirely based on a computational model of the fusion construct, as there was no crystal structure for the C-terminal dArmRP-DARPin fusion available. As expected, this construct produced a ring-like structure, where the N- and C-terminal regions of the protein are covalently linked. The crystal structure agreed well with the designed model with an overall Cα RMSD of 4.5 Å, with the deviation mainly caused by the first DARPin. The N-terminal DARPin exhibited considerable flexibility corresponding to poorly defined electron density, but it efficiently shielded the binding surface. Not surprisingly, the flexible DARPin domain was not involved in strong crystal contacts. The electron density for the shared helix, on the other hand, was well defined and decreased gradually towards the N-terminus of the helix and the DARPin-domain.

The most important goal of our design was to observe peptide binding on the surface of the dArmRP without direct crystal contacts in the peptide binding area, which could interfere with binding of the peptide. Even though the peptide was shifted by one repeat, peptide binding was observed in a geometrically ideal fashion and found to be almost identical in four of five arginine residues and in all lysine pockets, as intended by the design. With an inter-repeat distance of 6.3–7.3 Å, the curvature of the dArmRP domain of the construct matched that of the most regular dArmRP-peptide complex structures solved so far^16^, except for the first repeat. There was no direct crystal contact to the binding pockets of the first repeat, but between the shared helix of a symmetry-related construct and the shared helix-dArmRP interface. This contact probably induced a lengthened inter-repeat distance of 7.34 Å between the first and the second repeat of the dArmRP domain. This small deviation from the ideal curvature (6.7–7 Å) is also the most likely cause for the shift of the peptide by one repeat. However, the fact that the remaining repeats bound four of the five arginines and that all five lysines were bound ideally confirmed the success of the design. The shielded binding surface led to the first dArmRP-peptide structure devoid of direct crystal contacts, which could have influenced peptide binding.

We have therefore established that, firstly, rigid fusions of DARPins and dArmRPs are possible and the design is as straightforward as for DARPin-DARPin fusions, with a high accuracy of the *in silico* models. Secondly, the validity of previously determined dArmRP structures, in which it was unclear to which extent the crystal contacts influenced peptide binding, has been confirmed. Although crystal contacts can have a major influence on peptide binding by displacing it, dArmRP scan crystallise on their own, maintaining their ideal equilibrium curvature and peptide binding. This finding is important for later design steps, in which new pocket and peptide combinations need to be analysed regularly and rapidly by crystallography.

For the design we used the Rosetta framework, because this program suite allows to generate and evaluate thousands of models *in silico*. Nevertheless, we observed significant deviations between the designs and the crystal structure, which is primarily a consequence of the solenoid character of the DARPin and dArmRP domains and the fusion strategy. Both DARPins and dArmRPs were initially designed using a consensus sequence approach with point mutations on the dArmRP domain to enable the fusion strategy. During the *fixbb* step in the *Rosetta* framework the side chain packing interactions in the hydrophobic core are slightly underestimated, since the main chain coordinates were held constant, which causes larger shifts during the subsequent *relax* step. Yet, we think that a more sophisticated design strategy, involving e.g. *backrub* minimisation or more rounds of flexible and constrained minimisation of the whole construct or parts of it could lead to a better agreement between the designs and the crystal structures. But despite all advances in the forcefield, this prediction of deviations will still be limited by the crystal forces, which play a huge role in the crystallisation of solenoid repeat proteins, especially when they are in such an elongated shape as shown here. Future designs will therefore still have to prove whether better minimisation and relaxation can increase the accuracy of the designs, but we are optimistic on that.

Furthermore, we think that the DARPin-dArmRP fusions will be valuable also for further applications in which geometrically predictable orientations of the paratopes will be of interest, besides applications in crystallography.

As an analogy, rigid DARPin-DARPin fusions were found to be highly useful in determining the conformation of the oncogenic HER2 receptor on the surface of cells^24^. In that study, the rigid fusion of a binding and a non-binding DARPin was used to exclude certain conformational states of the receptor that are accessible to other members of the EGFR family. Similarly, biparatopic DARPins, which are fusions of DARPins recognizing two different epitopes on neighbouring receptor molecules, have shown strong apoptosis-inducing effects on cancer cells overexpressing HER2^25^ as well as on c-MET^26^. We propose that also DARPin-dArmRP fusions may be useful in this context, as they may connect conformational epitopes (bound by the DARPin) with linear epitopes (bound by the dArmRP) in a rigid manner with predictable geometry. Such fusions can now be constructed with a high level of precision.

## Material and Methods

### Shared helix design

Similar to the DARPin-DARPin fusion designs described in ref.^21^, a shared helix connecting the DARPin and dArmRP termini was constructed with PyMol^27^ and the sequence of the interaction interface of the helix and the fusion partner were optimised using the Rosetta protein design suite^23^ with the *fixbb* module involving a *constrained relax* run before and a *full relax* run after the design step. Cα RMSDs between the designs and the crystal structures were calculated with the CEAlign plugin of PyMOL, using only the Cα-carbon atoms with 0 cycles of refinement.

### Cloning, expression and purification

The DNA for the shared helix designs was ordered from Invitrogen GeneArt (Thermo Fisher Scientific, Massachusetts, USA) and inserted between the N-terminal DARPin and the C-terminal dArmRP by using a KasI and a Bsu36I restriction site. The constructs were cloned into the vector p148_3C (ref.^14^) or pQIq (ref.^28^) carrying an ampicillin resistance. Both vectors are derivatives of the pQE30 (Qiagen, Hilden, Germany) vector with a 3C-protease cleavable N-terminal His_6_ or His_10_ tag, respectively. *E*. *coli* BL21-Gold or *E*. *coli* XL1-blue cells were made competent and transformed with the respective plasmid. Gene expression was achieved in two ways: Either cells were grown to an OD of 0.6–1 in 1 L of 2xYT medium, inoculated with 50 mL preculture, and the expression induced by adding 1 mM IPTG and continued for 4 h at 37 °C; or expression was achieved in 1 L autoinduction medium^29^, which was inoculated with 50 mL preculture, and cells were continued to grow for 16 h at 30 °C. Cells were harvested by centrifugation at 5,000 x g for 10–15 min, resuspended in lysis and wash buffer (50 mM Tris/HCl pH 8, 500 mM NaCl, 20 mM imidazole) and lysed by sonication. To remove cell debris and non-lysed cells, the resuspension was centrifuged at 20,000 × g for 20 min and the supernatant loaded on a 5 mL Ni-NTA resin column (Qiagen, Hilden, Germany). The column was washed with 5 column volumes (cv) of washing buffer and protein was eluted using 3 cv lysis buffer supplemented with 250 mM imidazole. 2% (w/w) 3C protease, stored in 10 mM HEPES/NaOH pH 8, 150 mM NaCl, 1 mM DTT, 10 mM EDTA, 20% glycerol, was added and the imidazole removed by dialysis at 4 °C overnight against 50 mM Tris/HCl pH 8, 300 mM NaCl. To remove the protease and the His-tag, the dialysed elution fraction was loaded again on a 5 mL Ni-NTA resin column and the flow-through was collected and concentrated to 3–5 mL using Amicon centrifugal concentrators (30,000 MWCO, Merck Millipore, Massachusetts, USA). The concentrated protein was loaded on a Superdex S75 or S200 column (GE Life Sciences, Little Chalfont, Buckinghamshire, UK). Protein was eluted using 10 mM Tris/HCl pH 8, 100 mM NaCl at a flow-rate of 1 mL/min.

### Crystallisation and structure determination

Proteins were concentrated to 20 mg/mL with an Amicon centrifugal concentrator (30,000 MWCO, Merck Millipore, Massachusetts, USA) and set up for crystallisation. For the constructs without a peptide fusion a 2-fold molar excess of (KR)_5_ peptide was added prior to crystallisation. Suitable crystallisation conditions were screened with 96-well sparse matrix screens at 4 °C (Hampton Research, California, USA and Molecular Dimensions (Anatrace, Ohio, USA)). For each condition, three different mother liquor to protein ratios were used (1:1, 2:1, 3:1 or 5:1) in 300–400 nL drops, equilibrated against 75 µL of reservoir solution. Initial hits were fine-screened using a vertical pH and a horizontal precipitant gradient.

Crystals were flash-frozen in liquid nitrogen after incubation for 10 s in mother liquor supplemented with 20% ethylene glycol. For data collection, crystals were taken to beamlines X06DA or X06SA (Paul Scherrer Institute, Villigen, Switzerland) equipped with an Eiger 16 M detector or a Pilatus 2 M detector (Dectris, Baden-Wättwil, Switzerland) and data were collected at a wavelength of 1 Å. Crystallisation conditions and data collection and refinement statistics are summarized in SI Table 1. Data processing was done using XDS, XSCALE and XDSCONV^30^. Molecular replacement was used to solve the phases using PHASER^31^ and model building was carried out in Coot^32^. Refinement was done using REFMAC5^33^, PHENIX refine^34^ and BUSTER^35^. For determining the final resolution of the datasets, paired refinement was done in pdb_redo^36^ based on ref.^37^.

## Supplementary information

Supplementary information

## References

[1] Jost C, Plückthun A (2014). Engineered proteins with desired specificity: DARPins, other alternative scaffolds and bispecific IgGs. Curr. Opin. Struct. Biol..

[2] Simeon R, Chen Z (2018). *In vitro*-engineered non-antibody protein therapeutics. Protein Cell.

[3] Huber AH, Weis WI (2001). The structure of the beta-catenin/E-cadherin complex and the molecular basis of diverse ligand recognition by beta-catenin. Cell.

[4] Conti E, Kuriyan J (2000). Crystallographic analysis of the specific yet versatile recognition of distinct nuclear localization signals by karyopherin α. Structure.

[5] Conti E, Uy M, Leighton L, Blobel G, Kuriyan J (1998). Crystallographic analysis of the recognition of a nuclear localization signal by the nuclear import factor karyopherin alpha. Cell.

[6] Parmeggiani F (2008). Designed armadillo repeat proteins as general peptide-binding scaffolds: consensus design and computational optimization of the hydrophobic core. J. Mol. Biol..

[7] Alfarano P (2012). Optimization of designed armadillo repeat proteins by molecular dynamics simulations and NMR spectroscopy. Protein Sci..

[8] Madhurantakam C, Varadamsetty G, Grütter MG, Plückthun A, Mittl PRE (2012). Structure-based optimization of designed armadillo-repeat proteins. Protein Sci..

[9] Varadamsetty G, Tremmel D, Hansen S, Parmeggiani F, Plückthun A (2012). Designed Armadillo Repeat Proteins: library generation, characterization and selection of peptide binders with high specificity. J. Mol. Biol..

[10] Hansen S (2016). Structure and energetic contributions of a designed modular peptide-binding protein with picomolar affinity. J. Am. Chem. Soc..

[11] Reichen C, Hansen S, Plückthun A (2014). Modular peptide binding: from a comparison of natural binders to designed armadillo repeat proteins. J. Struct. Biol..

[12] Ernst P, Plückthun A (2017). Advances in the design and engineering of peptide-binding repeat proteins. Biol. Chem..

[13] Reichen C (2016). Structures of designed armadillo-repeat proteins show propagation of inter-repeat interface effects. Acta Crystallogr. Sect. D, Struct. Biol..

[14] Reichen C, Madhurantakam C, Plückthun A, Mittl PRE (2014). Crystal structures of designed armadillo repeat proteins: Implications of construct design and crystallization conditions on overall structure. Protein Sci..

[15] Hansen S, Kiefer JD, Madhurantakam C, Mittl PRE, Plückthun A (2017). Structures of designed armadillo repeat proteins binding to peptides fused to globular domains. Protein Sci..

[16] Hansen S (2017). Curvature of designed armadillo repeat proteins allows modular peptide binding. J. Struct. Biol..

[17] Binz HK, Stumpp MT, Forrer P, Amstutz P, Plückthun A (2003). Designing repeat proteins: well-expressed, soluble and stable proteins from combinatorial libraries of consensus ankyrin repeat proteins. J. Mol. Biol..

[18] Binz HK (2004). High-affinity binders selected from designed ankyrin repeat protein libraries. Nat. Biotechnol..

[19] Plückthun A (2015). Designed ankyrin repeat proteins (DARPins): binding proteins for research, diagnostics, and therapy. Annu. Rev. Pharmacol. Toxicol..

[20] Batyuk A, Wu Y, Honegger A, Heberling MM, Plückthun A (2016). DARPin-based crystallization chaperones exploit molecular geometry as a screening dimension in protein crystallography. J. Mol. Biol..

[21] Wu Y (2017). Rigidly connected multispecific artificial binders with adjustable geometries. Sci. Rep..

[22] ElGamacy M (2018). An interface-driven design strategy yields a novel, corrugated protein architecture. ACS Synth. Biol..

[23] Leaver-Fay A (2011). ROSETTA3: an object-oriented software suite for the simulation and design of macromolecules. Methods Enzymol..

[24] Jost C (2017). Rigidity of the extracellular part of HER2: Evidence from engineering subdomain interfaces and shared-helix DARPin-DARPin fusions. Protein Sci..

[25] Tamaskovic R (2016). Intermolecular biparatopic trapping of ErbB2 prevents compensatory activation of PI3K/AKT via RAS–p110 crosstalk. Nat. Commun..

[26] Andres F (2019). Inhibition of the MET kinase activity and cell growth in MET-addicted cancer cells by bi-paratopic linking. J. Mol. Biol..

[27] DeLano, W. L. The PyMOL Molecular Graphics System. *Schrödinger LLC*, Version 1, http://www.pymol.org (2002).

[28] Simon M, Zangemeister-Wittke U, Plückthun A (2012). Facile double-functionalization of designed ankyrin repeat proteins using click and thiol chemistries. Bioconjug. Chem..

[29] Studier FW (2005). Protein production by auto-induction in high density shaking cultures. Protein Expr. Purif..

[30] Kabsch W (2010). XDS. Acta Crystallogr. Sect. D Biol. Crystallogr..

[31] McCoy AJ (2007). Phaser crystallographic software. J. Appl. Crystallogr..

[32] Emsley P, Lohkamp B, Scott WG, Cowtan K (2010). Features and development of Coot. Acta Crystallogr. Sect. D Biol. Crystallogr..

[33] Murshudov GN (2011). REFMAC 5 for the refinement of macromolecular crystal structures. Acta Crystallogr. Sect. D Biol. Crystallogr..

[34] Afonine PV (2012). Towards automated crystallographic structure refinement with phenix.refine. Acta Crystallogr. Sect. D Biol. Crystallogr..

[35] Bricogne, G. *et al*. BUSTER 2.10.3. *Cambridge*, *United Kingdom Glob*. *Phasing Ltd*. (2017).

[36] Joosten RP, Joosten K, Murshudov GN, Perrakis A (2012). PDB_REDO: constructive validation, more than just looking for errors. Acta Cryst.

[37] Karplus PA, Diederichs K (2012). Linking crystallographic model and data quality. Science.

