# Supplementary material for: Rigid fusions of designed helical repeat binding proteins efficiently protect a binding surface from crystal contacts
Source: Sci Rep. 2019 Nov 7;9:16162. doi: 10.1038/s41598-019-52121-9 (PMC6838082; doi:10.1038/s41598-019-52121-9)
Supplement: Supplementary file 1 — Supplementary information [file 41598_2019_52121_MOESM1_ESM.pdf]

Supplementary Material

for

Rigid fusions of designed helical repeat binding proteins efficiently protect a binding surface from crystal contacts

Authors Patrick Ernst[a], Annemarie Honegger[a], Floor van der Valk[a], Christina Ewald[a,b], Peer R. E. Mittl[a] and Andreas Plückthun[a, *]

Affiliations:

[a] Department of Biochemistry, University of Zürich, Winterthurerstrasse 190, 8057 Zürich, Switzerland

[b] Present address: Flow Cytometry Facility, University of Zürich, Winterthurerstrasse 190, 8057 Zürich, Switzerland

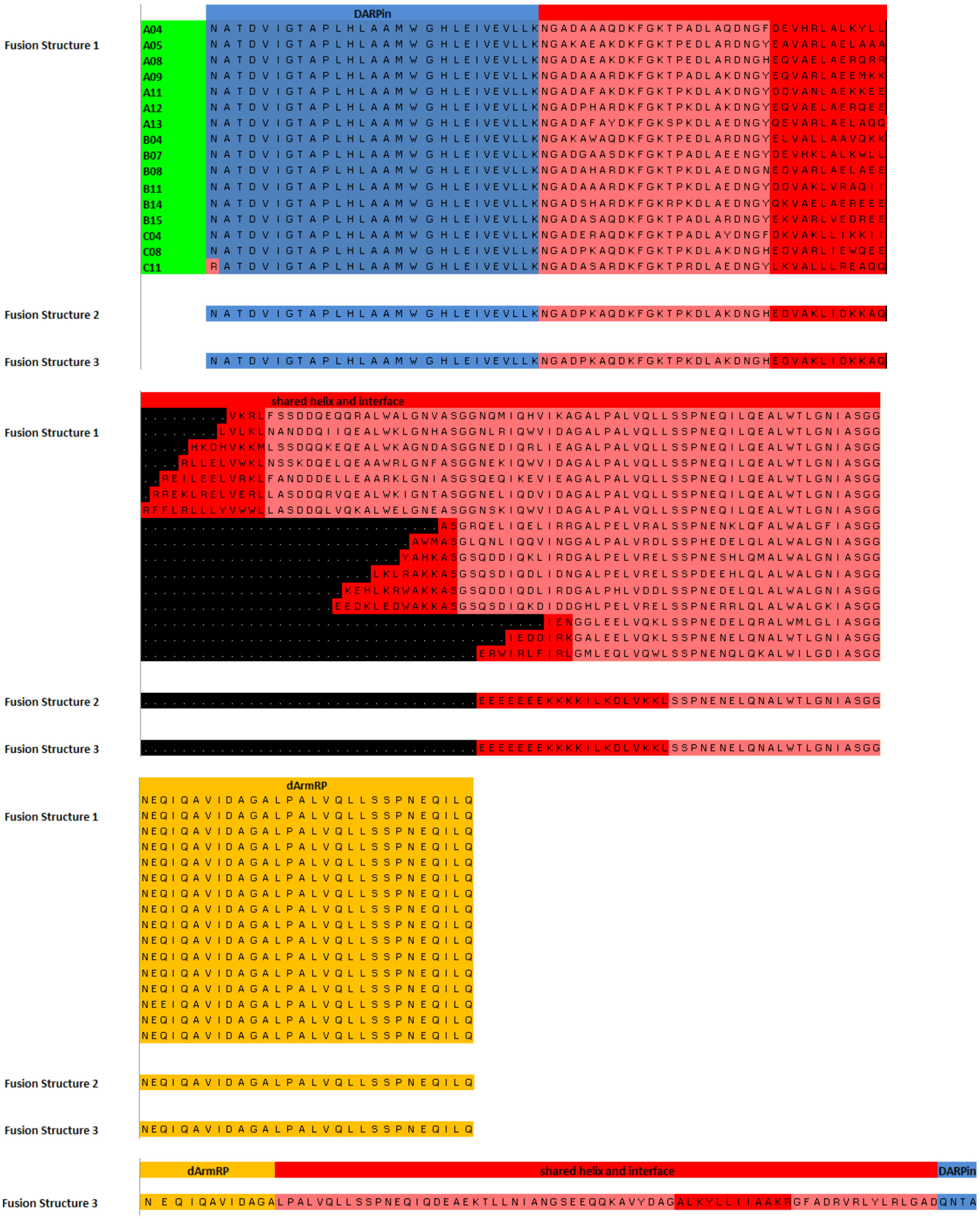

Supplementary Figure S1: Sequences of different fusions tested. Constructs denoted "A" are shared helices of H2, constructs denoted "B" are shared helices of H3 and constructs denoted "C" of H1. The DARPin part (only the end is shown) is in blue, shared helix in red, interface and single mutations in pink, dArmRP in orange, gaps in the alignment in black. For Fusion Structure 3 also the C-terminal shared helix is shown.

a

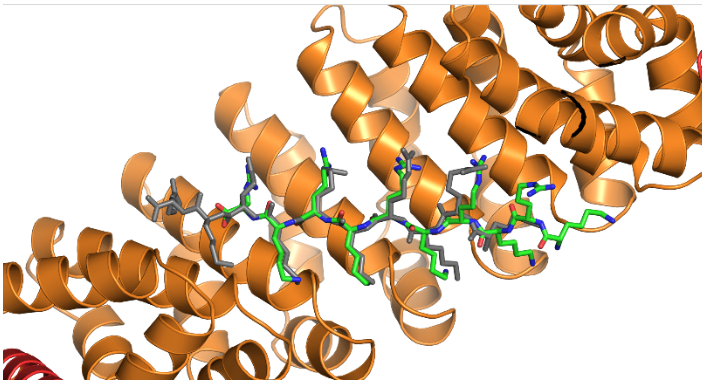

b

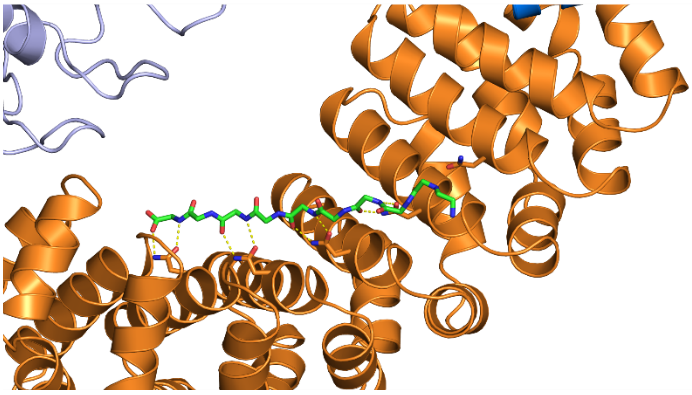

Supplementary Figure S2: Detailed view of peptide interaction on the ring-like construct. The dArmRP is colored in orange and the peptide in green. **a** Peptide register shift by one repeat, ideal peptide binding is shown in grey. **b** Backbone interactions of the peptide to the Asn37 ladder by bidentate hydrogen bonds (yellow).

Supplementary Table S1 - Data collection and refinement statistics for structures. Values in parentheses show the data for the highest resolution shell

	Fusion 1 (6SA6)	Fusion 2 (6SA7)	Fusion 3 (Ring, 6SA8)
**condition**	18.6 % PEG 6,000		25 % PEG 2000 MME
	200 mM $MgCl_2$	4.3 M NaCl	0.2 M $MgCl_2$
	0.1M Tris HCl pH 8.1	0.1M HEPES NaOH pH 7.5	0.1M Tris HCl pH 8.5
**Data collection**			
Resolution range	45.56 - 1.6 (1.657 - 1.6)	45.36 - 3.3 (3.418 - 3.3)	46.49 - 2.4 (2.486 - 2.4)
Space group	P21	$P2_1$	C2221
Cell dimensions			
a, b, c (Å)	57.08 51.56 67.72	54.47 167.54 95.03	92.97 145.46 115.62
α, β, γ (°)	90, 95.24, 90	90, 97.94, 90	90, 90, 90
Total Reflections	159331 (14444)	180178 (19206)	778723 (83722)
Unique reflections	49723 (4704)	24180 (2488)	30896 (3032)
Multiplicity	3.2 (3.1)	7.5 (7.7)	25.2 (27.6)
Completeness (%)	95.70 (91.47)	95.09 (97.84)	99.36 (100.00)
I/σ(I)	11.34 (0.97)	7.33 (1.18)	11.95 (1.16)
Wilson B-factor	25.96	101.47	54.37
Rmerge	0.05692 (1.086)	0.227 (1.878)	0.3695 (4.355)
Rmeas	0.06844 (1.313)	0.2448 (2.012)	0.3774 (4.436)
Rpim	0.0375 (0.7288)	0.09092 (0.7215)	0.0757 (0.8418)
CC1/2	0.997 (0.451)	0.993 (0.448)	0.998 (0.562)
Refinement			
R-work	0.1823 (0.3322)	0.2326 (0.3776)	0.2376 (0.3085)
R-free	0.2012 (0.3483)	0.2760 (0.3824)	0.2749 (0.3457)
RMS(bonds)	0.006	0.006	0.004
RMS(angles)	1.01	1.04	0.93
Ramachandran plot (%)			
Favoured	99.49	98.89	96.72
Allowed	0.51	1.11	2.98
Outliers	0	0	0.3
Rotamer outliers (%)	1.63	0.77	1.54
Average B-factor (Å2)	34.77	1223.18	82.51
Non-hydrogen atoms	3359	7458	5149
Protein	2985	7458	5034
Ligand	30	0	4
Water	344	0	111
